# ARIA Care Pathways 2019: Next-Generation Allergic Rhinitis Care and Allergen Immunotherapy in Malaysia

**DOI:** 10.3390/jpm13050835

**Published:** 2023-05-15

**Authors:** Amir Hamzah Abdul Latiff, Salina Husain, Baharudin Abdullah, Palaniappan Suppiah, Vincent Tan, Tang Ing Ping, Kent Woo, Yoke-Yeow Yap, Claus Bachert, Holger J. Schunemann, Anna Bedbrook, Wienczyslawa Czarlewski, Jean Bousquet

**Affiliations:** 1 Allergy & Immunology Centre, Pantai Hospital Kuala Lumpur, Jalan Bukit Pantai, Taman Bukit Pantai, Kuala Lumpur 59100, Malaysia; amirlatiff@gmail.com; 2 Department of Otorhinolaryngology—Head & Neck Surgery, Universiti Kebangsaan Malaysia Medical Centre, Jalan Yaacob Latif, Bandar Tun Razak, Kuala Lumpur 56000, Malaysia; drsalina_h@yahoo.com; 3 Department of Otorhinolaryngology—Head and Neck Surgery, School of Medical Sciences, Universiti Sains Malaysia, Kubang Kerian 16150, Malaysia; 4 Otorhinolaryngology, Gleneagles Hospital Penang, 1, Jalan Pangkor, George Town 10050, Malaysia; palani.ent@gmail.com; 5 Otorhinolaryngology, KPJ Klang Specialist Hospital, Persiaran Rajawali, Bandar Baru Klang, Klang 41150, Malaysia; entdrvincenttan@gmail.com; 6 Department of Otorhinolaryngology—Head and Neck Surgery, Faculty of Medicine & Health Sciences, University Malaysia Sarawak, Kota Samarahan 94300, Malaysia; ingptang@yahoo.com; 7 Allergy & Immunology Clinic, Gleneagles Hospital Kuala Lumpur, Jalan Ampang, Kampung Berembang, Kuala Lumpur 50450, Malaysia; allergywoo@gmail.com; 8 Otorhinolaryngology, KPJ Johor Specialist Hospital, 39B Jalan Abdul Samad, Johor Bahru 80100, Malaysia; yokeyeow@gmail.com; 9 Department of Otorhinolaryngology—Head and Neck Surgery, University Hospital of Münster, 48149 Münster, Germany; claus.bachert@ugent.be; 10 International Airway Research Center, First Affiliated Hospital, Sun Yat-Sen University, Guangzhou 510080, China; 11 Upper Airways Research Laboratory, Faculty of Medicine, Ghent University, 9000 Ghent, Belgium; 12 Department of Clinical Epidemiology and Biostatistics and Medicine, McMaster University, Hamilton, ON L8S 4K1, Canada; schuneh@mcmaster.ca; 13 ARIA & MASK-air, 34090 Montpellier, France; anna.bedbrook@inserm.fr (A.B.); wieniaczarlewski@gmail.com (W.C.); 14 Medical Consulting Czarlewski, 92300 Levallois, France; 15 Institute of Allergology, Charité—Universitätsmedizin Berlin, Corporate Member of Freie Universität Berlin, Humboldt-Universität zu Berlin, 13353 Berlin, Germany; jean.bousquet@orange.fr; 16 Fraunhofer Institute for Translational Medicine and Pharmacology ITMP, Allergology and Immunology, 12203 Berlin, Germany; 17 University Hospital of Montpellier, University of Montpellier, 34000 Montpellier, France; 18 Inserm Equipe d’Epidémiologie Respiratoire Intégrative, CESP, 94807 Villejuif, France

**Keywords:** allergic rhinitis and its impact on asthma, clinical guidelines, integrated care pathways, intranasal corticosteroids, antihistamines, allergen immunotherapy

## Abstract

An increase in the prevalence of allergic rhinitis (AR) worldwide presents a significant burden to the health care system. An initiative was started in Europe designated as Allergic Rhinitis and Its Impact on Asthma (ARIA) to develop internationally applicable guidelines by utilising an evidence-based approach to address this crucial issue. The efforts are directed at empowerment of patients for self-management, the use of digital mobile technology to complement and personalise treatment, and establishment of real-life integrated care pathways (ICPs). This guideline includes aspects of patients’ and health care providers’ management and covers the main areas of treatment for AR. The model provides better real-life health care than the previous traditional models. This review summarises the ARIA next-generation guideline in the context of the Malaysian health care system.

## 1. Introduction

Allergic rhinitis (AR) is a worldwide health problem that is particularly prevalent in Southeast Asia [1]. According to an Asia Pacific survey, 19.8% of children aged 13–14 years were impacted, with 8.7% of these children suffering with AR [2]. According to a Malaysian study, the total prevalence of AR symptoms in the paediatrics population was 27%, with a significantly higher prevalence in the 12 to 14-year-old age group (38.2%) than the 5 to 7-year-old age group (18.2%) [3]. Allergic march is a trend of atopic dermatitis and food allergies in babies that progresses to chronic respiratory problems in school-aged children, including AR and asthma [4]. In Malaysia, 80% of people with AR are hypersensitive to aeroallergens such as pollen and dust [5].

AR is typically caused by perennial or seasonal allergies to the indoor and/or outdoor environment. House dust mites, pets, cockroaches, and mould are the primary causes of perennial AR, whereas grass, trees, and weeds are the primary causes of seasonal AR. House dust mites (HDMs), particularly Dermatophagoides pteronyssinus (Der p) and Dermatophagoides farina (Der f), are the most common indoor allergens for allergic diseases such as AR and asthma worldwide. Malaysia’s tropical climate, which is warm and humid all year, promotes the growth of dust mites and mould. The Allergic Rhinitis and Its Impact on Asthma (ARIA) next-generation recommendations and integrated care pathways (ICPs) for AR in reference to the Malaysian health care system are reviewed in the paper [6,7]. These ICPs cover all elements of patient care and provide direction to health care providers in critical areas of AR treatment.

## 2. Management of AR in the Health Care System of Malaysia

In Malaysia, both primary and secondary health care systems deal with AR patients, which are further separated into public and private (commercial) organisations [8,9]. In addition to commercially owned pharmacies, primary health care is provided at government health clinics that charge a minimal or no cost, or by general practitioners, who are doctors in private clinics that charge a greater remuneration for their services. The Health Ministry of Malaysia manages local health clinics in both urban and rural regions that are staffed by doctors or paramedics. The majority of private clinics, which are mostly situated in urban or suburban areas, are run by general practitioners or other doctors hired by the owners. Similarly, dual health care frameworks (public and private health care) apply to secondary care. Medical specialists in hospitals typically provide secondary health care. Patients may be referred from primary care to secondary care by health care providers, or they may self-refer if they require additional consultation. The former is appropriate to public institutions, while the latter is applicable to private organisations; nonetheless, one arrangement does not always preclude the other. The majority of AR patients are expected to be handled and managed in primary care, with difficult cases being managed in secondary care. By incorporating next-generation AR guidelines into the local health care system, a consistent approach to managing AR may be adopted despite the varying level of training, knowledge, and experience.

Community pharmacists are frequently the first responders for AR patients’ needs because of the availability of over-the-counter oral antihistamines and patients’ reluctance to seek medical consultation [10]. They serve as an important health care resource, facilitating patient–physician dialogue. It is critical for community pharmacists to diagnose and treat AR because they can play a key role in medication adherence, for example, counselling patients while OTC (over-the-counter) medicines are supplied. In severe circumstances, patients may be referred to a general practitioner (GP) or a health care specialist for additional treatment.

Patients with AR who do not respond to over-the-counter antihistamines frequently seek additional treatment from their general practitioners (GPs). GPs must be well-informed and knowledgeable in order to provide an accurate AR diagnosis and provide suitable treatment. It should be noted that not all health care practitioners are familiar with the proper administration of intranasal corticosteroid sprays. Incorrect administration can result in suboptimal effects, which can lead to patient frustration and poor compliance. Despite the fact that various international recommendations for AR have been published, their impact and effectiveness on GPs’ management of AR patients is unknown. ARIA Care Pathways 2019 is a useful practical guideline that assists frontline clinicians like GPs and pharmacists in identifying and managing patients with AR, as well as advising them on the necessity for referral in cases with refractory and complicated rhinitis.

## 3. Next-Generation ARIA-GRADE Guidelines

The goal of selecting medication for AR patients is to keep the patient’s condition under control. With the ongoing support of many organisations such as MASK (Mobile Airways Sentinel Network) and POLLAR (Impact of Air Pollution on Asthma and Rhinitis, EIT Health) and in collaboration with professional and patient organisations in the field of allergy and respiratory diseases, a meeting on chronic disease care was held in Paris on 3 December 2018, where a new project (the ARIA guidelines) was launched [11,12]. It was suggested that real-life ICPs be used to create digitally enabled, integrated, person-centred therapy for rhinitis and asthma multimorbidity encompassing environmental exposure [11].

**Figure 1:**
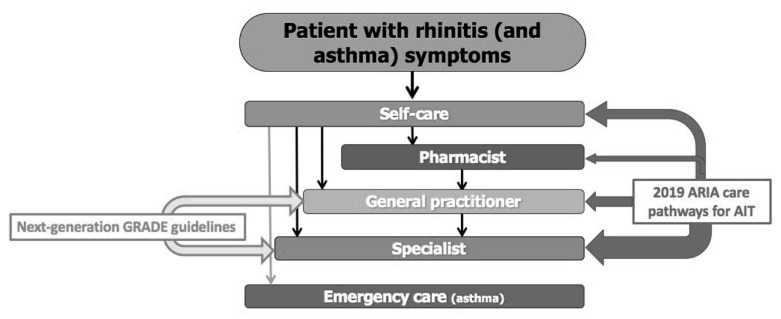
A summary of the next-generation ARIA care pathways [6]. Reprinted/adapted with permission from Ref. [6]. 2019, Bousquet et al.

ICPs are interdisciplinary care plans that outline critical treatment milestones for patients. They promote the translation of guidelines, their incorporation into local health care systems, and their use in clinical practise. The formation of AIRWAYS ICPs was the initial step towards the development of ICPs for the multimorbidity of rhinitis and asthma [13]. Figure 1 depicts a summary of the care pathways.

Although the Grading of Recommendations Assessment, Development, and Evaluation (GRADE) technique clearly considers all types of study designs, guideline developers usually prefer to confine guidelines to randomised controlled trials (RCTs). GRADE also considers evidence on values and preferences, as well as acceptability, feasibility, and directness of results. Real-world data (RWD) is increasingly being used to inform health care practise. Quintessentially, both GRADE and RWD can be combined to generate real-world evidence (RWE).

During the Paris meeting, next-generation guidelines for the pharmacologic treatment of AR were developed, which were tested utilising RWD provided by mobile technology and chamber studies [11,12,14,15,16,17,18]. These suggestions were used to improve the MASK algorithm for AR treatment presented by a consensus committee.

## 4. Development of ARIA Integrated Care Pathways for Pharmacologic Treatment

**Figure 2:**
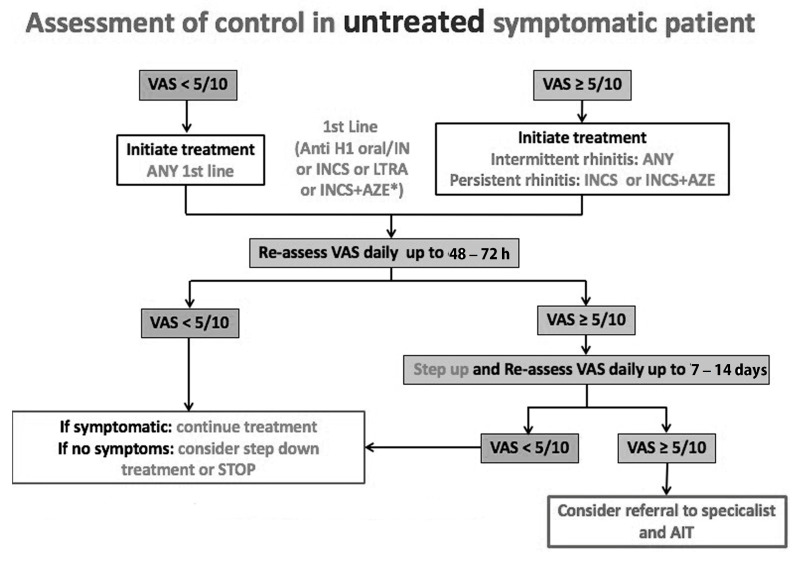
The step-up algorithm in untreated patients using the visual analogue scale (adolescents and adults) [19]. The proposed algorithm helps determine the therapy steps and the visual analogue scale levels of the patient’s preferences. If ocular problems persist after starting medication, add intra-ocular treatment. * Consider INS + AZE if previous treatment is ineffective. Reprinted/adapted with permission from Ref. [19]. 2016, Bousquet et al.

**Figure 3:**
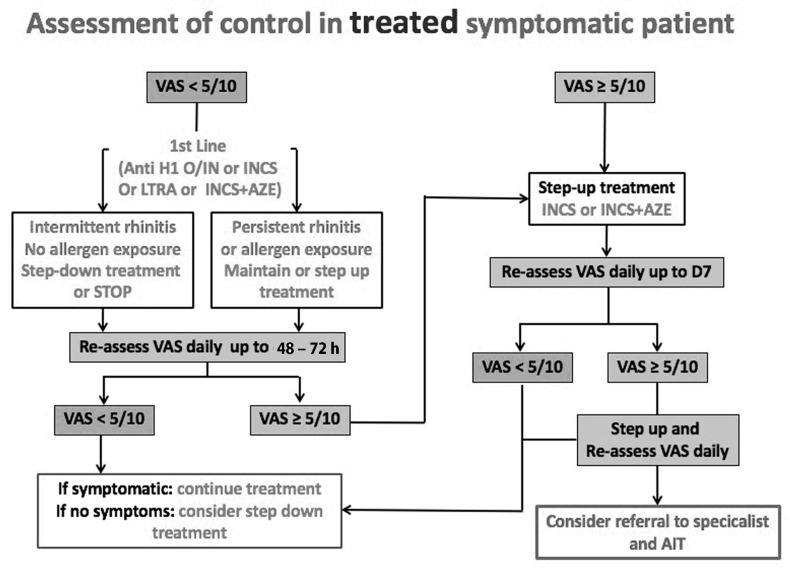
The step-up algorithm in treated patients using the visual analogue scale (adolescents and adults) [19]. The proposed algorithm considers the treatment steps and the patient’s preference visual analogue scale levels. If ocular symptoms remain following treatment, add intra-ocular treatment. Reprinted/adapted with permission from Ref. [19]. 2016, Bousquet et al.

The MASK algorithm, which is based on the visual analogue scale (VAS), was developed and digitalised to suggest step-up or step-down AR treatment that may be adjusted and altered using Malaysian medicinal products and resources (Figure 2 and Figure 3).

Although there have been few head-to-head drug comparisons utilising RCTs, AR medication comparisons have been advocated by reviews and guidelines [14,15,16,19]. Most AR medications have a comparable impact, according to a Health Technology Assessment [20]. However, this study employed an overly restrictive method that did not allow for medication-distinct characteristics.

The ARIA revision 2016 and the US Practise Parameters 2017 were created independently, but both used the same GRADE-based methodological approach [15,16]. Interestingly, the same questions were addressed. In the treatment of moderate-to-severe rhinitis, two major outcomes were considered: efficacy and speed of action, which resulted in comparable recommendations [15,16]. All of these recommendations had a low or very low level of evidence. Both the ARIA 2016 revision and the US Practise Parameters 2017 recommendations, which are mostly based on RCT findings, support the MASK methodology [15,16,19].

To investigate the commencement of action of AR medications, traditional Phase III double-blind RCTs using both park setting and allergen exposure chamber (AEC) trials can be used [21,22]. AECs offer the advantage of being able to evaluate medication efficacy in minutes.

In the Ontario and Vienna chambers, several oral and intranasal medications were tested. The Ontario Chamber studies indicated that azelastine and its combinations, including MPAzeFlu, have a rapid onset of efficacy. Other intranasal H1-antihistamines take longer to have an effect. Intranasal corticosteroids (INCS), either alone or in conjunction with oral H1-antihistamines, are ineffective within 2 h of administration. In Vienna chamber tests, azelastine and levocabastine with fluticasone responded faster than oral H1-antihistamines or INCS [23].

The MASK algorithm as well as the next-generation ARIA guidelines were validated or refined by combining GRADE recommendations with RWD and data from mHealth technologies [23]. Despite the availability of numerous mHealth technologies for AR, only MASK has published information on medications that can be used in RWE [11,24]. The findings of the RWE research for the treatment of AR are depicted in Box 1.

Box 1 **Results of real-world evidence for the treatment of allergic rhinitis [23]. Reprinted/adapted with permission from Ref. [23]. 2020, Bousquet et al.**

1. Patients did not follow guidelines and often self-medicate.

2. Adherence to treatment was poor.

3. Patients treat themselves as and when needed, depending on the control of their disease, and increase their treatment when they are unwell. However, co-medication does not improve the control.

4. MPAzeFlu is superior to INCS, which are superior to oral H1-anti-histamines.

Although direct verification of adherence is hard because MASK users may not input data every day and may not record all drugs consumed, secondary adherence was determined to be less than 5% using modified the Medication Possession Ratio (MPR) and Proportion of Days Covered (PDC) [25]. Potential biases in participatory data research include the likelihood of sample bias, result misclassification, and the availability of extremely limited information on patient (or day) characteristics due to ethical concerns. The app’s users do not represent all rhinitis patients. MASK used days in a cross-sectional analysis because there is no obvious treatment pattern and a long-term study was not feasible because users normally use the app on occasion [11].

Although a physician’s diagnosis of AR was not validated, the majority of users are likely to have rhinitis (allergic or non-allergic) [11]. Nonetheless, mobile technology is becoming a crucial tool for better understanding and managing AR by providing previously unavailable information. In the treatment of pollen-induced AR, there is a complete disconnect between the physician’s prescription and the patient’s behaviour. The vast majority of doctors prescribe medications for the entire season, suggesting patients take them on a regular basis even if they are not suffering any symptoms. However, the vast majority of patients take their drugs on demand, resulting in insufficient AR control and failure to adhere to guidelines. This is a frequent practise in Malaysia and its bordering countries [26]. Interestingly, physicians do not follow AR prescriptions when they have it, mirroring the conduct of patients they have treated.

## 5. Next-Generation ARIA-GRADE Guidelines Algorithm

In the next-generation ARIA-GRADE guidelines, the algorithm presented a stepwise approach for selecting AR medicine based on GRADE recommendations optimised using RWE and chamber studies [13,27]. The proposed technique verifies the majority of GRADE recommendations for AR, allows RWE to support some conditional evidence, and provides some fresh insights. The efficacy of combined oral H1-antihistamines and INCS was not shown to be superior to INCS alone. The combined efficacy of intranasal H1-antihistamines and INCS was shown to be more substantial than that of INCS alone. Intranasal H1 antihistamine medications work in minutes. When other treatments fail to control symptoms, the greater costs of a fixed combination of INCS and intranasal H1-antihistamines are justified [14]. In general, the GRADE guideline recommendations concur on a few essential criteria for pharmacotherapy (Box 2) [14,15,16,19].

Box 2 **Key points of the recommendations for pharmacotherapy [23]. Reprinted/adapted with permission from Ref. [23]. 2020, Bousquet et al.**

1. Intranasal corticosteroids are potent medications but require a few hours to days to be effective.

2. Intranasal H1-antihistamines are less effective than intranasal corticosteroids but have faster onset of action (within minutes).

3. Though oral H1-antihistamines are less potent than intranasal corticosteroids, most patients prefer oral to intranasal medications.

4. There is no advantage of using combined oral H1-antihistamines and intranasal corticosteroids over intranasal corticosteroids alone.

5. The combined efficacy of intranasal H1-antihistamines and intranasal corticosteroids is superior to intranasal corticosteroids alone with rapid onset of action (within minutes). MPAzeFlu, which combines intranasal fluticasone propionate and azelastine, is effective in severe allergic rhinitis following failure of intranasal corticosteroids and can be given to patients who want faster symptomatic relief.

6. The effectiveness of intranasal corticosteroids is superior to leukotriene antagonists.

7. The recommended pharmacotherapy is safe at the usual prescribed dose.

8. Avoid the use of the sedating first-generation oral H1-antihistamines and prolonged application of topical vasoconstrictors.

9. Intramuscular corticosteroids have no role in the treatment of allergic rhinitis.

## 6. Allergen-Specific Immunotherapy

Allergen-specific immunotherapy (AIT) is a well-known treatment for AR and/or asthma that can be given sublingually (SLIT) or subcutaneously (SCIT) [14,28,29,30,31,32,33]. The efficacy demonstrated in double-blind, placebo-controlled, randomised clinical trials (DB-PC-RCT) has been validated in prescription database research, which correlates to real-world experience [34]. Because AIT is more expensive in Malaysia than other AR or asthma therapy interventions, it should be explored in patients as part of a stratified medicine approach [35]. Though several AIT guidelines have been developed, the evidence-based approach differs in that many are complex, and none use ICPs [14,28,29,30,31,32,33] developed by ARIA 2019 for the prescription of both SCIT and SLIT [36].

Prescription of AIT should be based on symptoms encountered during allergen exposure, evidence of sensitisation, and the availability of high-quality, standardised extracts. The use of AIT products must conform to regulatory requirements and be supported by proof of efficacy and safety. Extracted allergens should not be considered generics. There is no evidence that mixing different allergens has the same effect as delivering each allergen individually. This practise may result in a dilutional effect and allergen degradation. Only use combined allergen products represented by allergen sources from homogeneous groupings [37]. Patients frequently become sensitised to many allergens (polysensitisation), albeit not all of these sensitisations are clinically significant. In various countries, like Malaysia, named patient products are used to customise therapy for patients by promoting items that are excluded from European legislation on allergen extracts. However, this strategy demands appropriate confirmatory investigations and RWE.

## 7. Safety of Subcutaneous Immunotherapy (SCIT)

Redness and swelling at the injection site are usual (local) symptoms that occur quickly after or several hours after the injection. Systemic responses include sneezing, nasal congestion, and hives. Serious injection responses are exceedingly rare, but they must be treated right away. The majority of adverse reactions occur within 30 min of injection, underlining the importance of patients being monitored at their doctor’s office for at least 30 min following an injection.

## 8. Safety of Sublingual Immunotherapy (SLIT)

Oral allergen drops or pills provide a higher safety profile than SCIT. SLIT can be administered at home after the initial dose, which is done under the supervision of a physician. The majority of adverse effects are minimal (mouth itch, lip swelling, nausea) and go away on their own within a few days after starting therapy. The severity of local adverse effects is often determined by their duration and impact on quality of life [38]. Patients starting on SLIT pills in several countries, excluding Europe, are advised of the likelihood of serious allergic responses, and adrenaline auto-injectors are commonly prescribed.

## 9. Shared Decision Making in Deciding on AIT

To enable shared decision making (SDM), the patient’s point of view should be taken into account at all times. There are numerous real-world studies that evaluate AIT knowledge, perceptions, expectations, and satisfaction. However, AR patients frequently lack information, and enhanced communication is required to improve patient knowledge and satisfaction.

AIT patients must completely adhere to the treatment in order for it to be effective. Noncompliance with an AIT regimen, as well as premature termination, are common occurrences [39]. The rate of AIT adherence has yielded conflicting results, but it appears to be low [40]. A well-organised physician time plan enhances not only safety, but also allows for more frequent follow-up and better adherence to medications.

SDM should be used from a medico-legal standpoint, using current medical knowledge. It is the physician’s job to inform the patient about treatment alternatives, risks, and benefits in accordance with professional standards.

The vast majority of patients manage their AR on their own, with few interactions with their doctors. Community pharmacists are the most accessible health care providers to the general public, and one of the most common conditions they treat is AR [41]. AIT products are accessible in a variety of nations, and pharmacists must be knowledgeable about this treatment. Pharmacists can assist patients in understanding adherence, AIT requirements, and the risks and benefits of the medication.

In many countries, allergic disorders are first identified and treated at the primary care level. Primary care’s ease of access, accessibility, and holistic role in AR treatment and patient-centred SDM are paramount. Despite this, only a small percentage of GPs receive formal allergy training at the undergraduate or postgraduate level. SCIT can be administered in primary care, and while there are risks, these can be reduced when performed in an ideal primary care setting by trained GPs who carefully choose patients and are capable of managing systemic allergic reactions.

## 10. Patient Stratification in AIT

SDM plays a crucial role in AIT. AIT is an expensive therapy in Malaysia that should only be delivered to eligible individuals and must be prescribed by professionals. Furthermore, patients should understand whether AIT is covered by their health system or insurance company, and whether it will result in a partial or full out-of-pocket cost.

**Figure 4:**
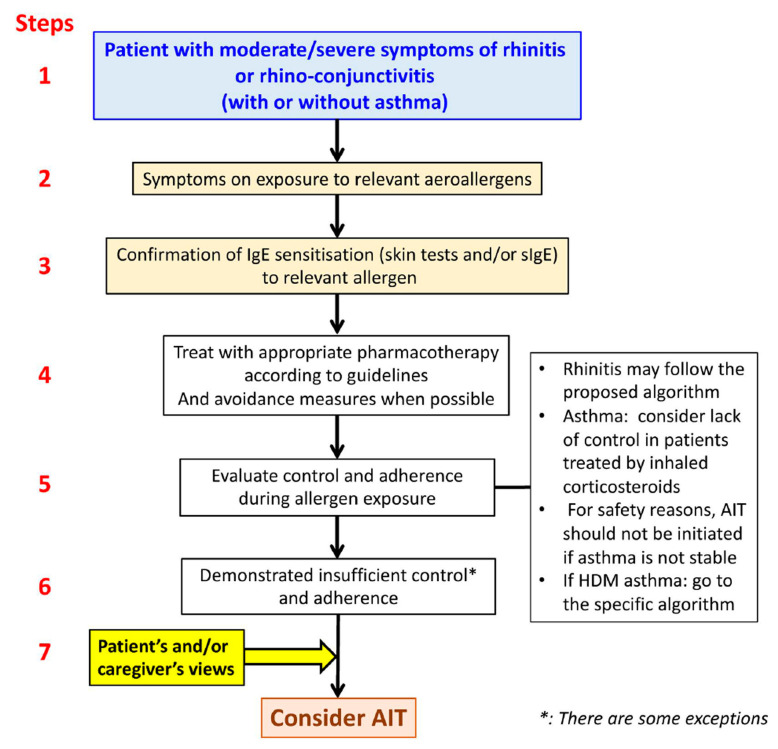
Flow of precision medicine for allergen immunotherapy [36]. Allergen-specific immunotherapy; (AIT). Reprinted/adapted with permission from Ref. [36]. 2019, Bousquet et al.

The ARIA 2019 care pathways advocate employing precision medicine when selecting an AIT regimen (Figure 4) [42]. In some situations, patients with pharmacologically managed AR, such as those who may suffer from thunderstorm-induced asthma, may be prescribed AIT [41]. AIT should be considered even in those with moderate AR, particularly (but not primarily) in those who have experienced asthma exacerbations during pollen season and live in geographically hazardous places. Except for the first-generation oral H1-antihistamines and intramuscular depot corticosteroids, which should be avoided, all of the medications listed are considered safe at the recommended dosage [37]. The committee for ‘fighting chronic illnesses for active and healthy ageing’ (MACVIA) developed a simple algorithm for step-up and step-down management (Figure 2) [19,20].

Patients with severe and/or uncontrolled asthma should not utilise AIT [43]. Biologics for severe asthma and AIT for allergic diseases are used to treat two distinct groups of people. There is currently no algorithm available for asthma. The Global Initiative for Asthma (GINA) has approved SLIT as a treatment for asthma induced by house dust mites [44]. According to the summary of product characteristics for the approved SLIT house dust mite tablet [45], (i) the patient should not have had a severe asthma exacerbation within the previous three months of AIT initiation, (ii) treatment should be delayed in patients with asthma who are suffering from an acute respiratory tract infection until the infection is resolved, (iii) AIT is not indicated for the treatment of acute exacerbations, and patients should be informed of the need to seek medical attention immediately if their asthma suddenly worsens; and (iv) mite AIT should be used as an add-on therapy to controller medication at first, and reduction in asthma controllers should be done gradually under supervision and in accordance with management guidelines. In the European Union, no other AIT medicine has been approved as a primary treatment for asthma.

The coexistence of numerous allergic disorders in the same patient, known as multimorbidity, is exceedingly common in allergic diseases, with over 85% of asthma patients also having AR. In comparison, only 20–30% of AR patients suffer asthma. AR multimorbidity worsens asthma severity [46]. AIT can control AR, conjunctivitis, and asthma multimorbidity. In the settings and permission of SLIT mite tablet use, multimorbidity was recognised as an indication for mite SLIT [45]. AIT is well tolerated by children and may have long-term effects after withdrawal [47,48]. A recent SLIT trial, a previous grass pollen SCIT study, and a meta-analysis have all demonstrated that AIT can help children with rhinitis to delay or avoid the onset of asthma [49,50,51]. A meta-analysis discovered that the chance of acquiring asthma in the short term is reduced while having no long-term effect [52]. The reduction, while little, is encouraging. As a result, AIT can be initiated in children who have moderate/severe AR and are not responding to medication. While more study is needed for an unequivocal indication, the prospect of preventing the formation of asthma in such children should be explored [2].

Older allergy sufferers have different immunologic and allergic characteristics than middle-aged people. Based on the available data, AIT may be beneficial in this demographic group [53].

## 11. mHealth in the AIT Precision Medicine Approach

Electronic diaries created by mobile phones or other mHealth tools can aid in patient identification [27,54]. Physicians can determine (i) whether moderate-to-severe uncontrolled disease is present, (ii) whether symptoms are associated with pollen season or other allergen exposure, (iii) whether pharmacologic treatment is adhered to, (iv) the duration of uncontrolled symptoms, and (v) the impact on work or school productivity based on a single year of survey. An electronic clinical decision support system could aid in the classification of patients for AIT in the future [55]. A similar approach can be offered for evaluating the efficacy of AIT throughout patient follow-up [51].

## 12. Challenges and Opportunities for Malaysia

In contrast to other parts of the world, Malaysia still has limited information on attitudes and practises related to the management of AR. Antihistamines, particularly the second-generation oral antihistamines, are still the mainstay of AR treatment in primary care, according to a recent survey done in Malaysia and other Southeast Asian (ASEAN) nations [26]. Notably, the survey revealed that some primary care practitioners continue to suggest first-generation oral antihistamines to AR patients. Another investigation [56] conducted in the Philippines to analyse both specialists’ and general practitioners’ practises in the treatment of AR patients yielded similar results. In the latter study, monotherapy with oral antihistamines was determined to be the preferable treatment option for mild AR. For moderate-to-severe patients, both monotherapy and combination therapy with leukotriene receptor antagonists, antihistamines, and INCS were favoured. The selection of such therapy regimens was mostly influenced by their efficacy and cost-effectiveness [26,56]. Cost-effectiveness is especially important in developing ASEAN nations with relatively limited public health care support and wherever patients must pay out of their own pockets. Because first-generation oral antihistamines are often less expensive than second-generation oral antihistamines, they quickly become the preferred option. Contrariwise, the Current Allergic Rhinitis Experiences Survey (CARES) [57], which evaluated the management practises of primary health care professionals (HCPs) in North America, found that more than 80% of HCPs regarded intranasal corticosteroids as the gold standard for the treatment of AR.

Medication adherence is crucial in achieving positive outcomes in chronic airway disorders such as AR. Despite this, it has been found that treatment adherence in allergic airway ailments is inadequate [58]. A recent Malaysian study of AR patients treated with INCS revealed that medication adherence is 58.9% [59]. Patients’ reasons for non-adherence include fear of bad consequences, unpleasant adverse effects, poor response, forgetfulness, and taking too many prescriptions. Patients developed a dread of potential adverse effects from previous medication use or seeing friends or family members take medications that caused them. To avoid non-adherence, patients must be well-informed in the requirement of the medications, the expected outcomes of the therapy, and the nature of the adverse effects of each specific medication. Prescribers can help patients adhere to medication by dispelling their worries about their unique need and addressing their treatment concerns.

These shortcomings and related constraints must be addressed effectively. The Malaysian Society of Allergy and Immunology (MSAI), in collaboration with Malaysian experts, developed an expert consensus statement to rationalise the pharmacologic therapy of AR [1], based on the core concepts of ARIA care [24]. The consensus statement outlined strategies for using pharmacotherapy in primary and secondary care, as well as specific recommendations for use in children and adults. All of the expected common adverse effects of each medication are clearly stated, allowing patients to be counselled properly. In addition, MSAI has released a consensus statement on the recommendation for AIT use in Malaysia for the benefit of medical practitioners [60].

Although pharmaceutical treatment is the primary mode of treatment for AR, surgical intervention may be a beneficial adjuvant for recalcitrant nasal obstruction and coexisting chronic rhinosinusitis [61]. To treat AR, a few surgical procedures are available, including turbinate reduction surgery, septoplasty, and endoscopic sinus surgery. When the turbinates swell as a result of AR, they can block the nasal airways, resulting in chronic nasal obstruction. Endoscopic turbinoplasty, radiofrequency ablation, laser therapy, and submucosal excision are some of the techniques used in turbinate reduction surgery [62]. When the nasal septum is crooked or deviated, septoplasty is performed to straighten it since these conditions may worsen the symptoms of AR [63]. Chronic rhinosinusitis is often treated with endoscopic sinus surgery [64]. Any blockages or diseased tissue in the sinonasal region are removed during surgery, which can facilitate an easier intranasal use and delivery of topical spray. IgE monoclonal antibodies are a form of biologic drug that has been demonstrated to be useful in the treatment of AR [65]. These antibodies function by specifically targeting and neutralising IgE, a kind of antibody produced by the immune system in reaction to allergens. The aforementioned drugs are safe and effective for the treatment of moderate-to-severe AR, and they can be used as an alternative or in addition to established pharmacologic therapies. However, there is currently no approved licence in Malaysia for the use of biologics for AR [1].

The World Health Organisation has recognised digital technologies as an important tool for strengthening health systems and achieving universal health coverage [66]. Initially, mobile health technologies and apps were assumed to be primarily relevant in industrialised countries. Smartphones have become significantly more affordable as smartphone manufacturers compete, resulting in the rapid expansion in smartphone ownership around the world [67]. This will enable a shift from traditional health care delivery to a more efficient and integrated technique meant to establish a cost-effective and patient-centred management [68].

A patient-centred information and communication technology system, such as the MASK app, is available in the majority of European nations in an increasing assortment of languages [24,69,70]. It includes every medicinal product tailored to each country, as well as a VAS to assess control and treatment response. The use of MASK has allowed for the detection of intraindividual variability on a daily basis in individuals with allergic multimorbidity. In research utilising MASK, distinguishing individuals with only AR from those with rhinoconjunctivitis has given considerable proof in the management of their condition. The usage of an app to manage AR with comorbid conditions exemplifies this journey. Multimorbidity is widely recognised in allergic airway disorders [71]. Multimorbidity has an impact on the daily fluctuating symptoms, adds to the severity, and impairs one’s job and activities. Self-monitoring with an electronic diary, targeted feedback, and customised education for specific patients are some of the advantages of mobile technology. Their application could enable and supplement patients’ self-management in their daily lives. According to a review of related RCTs, text messaging via mobile phone enhances medication adherence in AR patients [72]. This method provides a cost-effective and practical choice for patients, especially in developing nations like Malaysia, where insufficient public transportation, a lack of infrastructure, and excessive costs are major concerns.

Mobile technology improves treatment adherence by personalising treatment based on the condition and needs of patients. For example, the daily intake of medication can be monitored using a smartphone video camera system for direct or indirect verification by health experts. Using this monitoring system, any challenges to the adherence to the offered treatment can be spotted, allowing patients to be counselled and treatment changes to be made as needed.

## 13. Conclusions

The ARIA strategy for AR based on RCTs, observational research RWE, and chamber studies has been deemed sufficient and useful in Malaysian health care practise. Patients’ understanding, knowledge, and compliance are improved as a result of ARIA’s efforts to empower them for self-management, mHealth, and health care system-ICP. Both pharmacotherapy and AIT are efficient options that can be used to treat patients with allergic disorders.

## Data Availability

No new data were created or analysed in this study. All data included in this review have been previously published. If further specific data are needed, they may be provided by the corresponding author upon reasonable request.
